# Accuracy of BISAP score in prediction of severe acute pancreatitis

**DOI:** 10.12669/pjms.35.4.1286

**Published:** 2019

**Authors:** Anum Arif, Farhat Jaleel, Khalid Rashid

**Affiliations:** 1 Dr. Anum Arif, MBBS, FCPS. Department of Surgery, Aga Khan University of Health Sciences Karachi, Pakistan; 2 Dr. Farhat Jaleel, MBBS, FCPS. Professor, Head of Surgical Unit 6, Civil Hospital Karachi, Dow University of Health Sciences, Karachi, Pakistan; 3 Dr. Khalid Rashid, MBBS, FCPS. Associate Professor, Department of Surgery, Jinnah Post-Graduate Medical Center, Jinnah Sindh Medical University, Karachi, Pakistan

**Keywords:** Severe Acute pancreatitis, Ranson’s score, BISAP score

## Abstract

**Objective:**

To determine the accuracy of BISAP score in comparison with Ranson’s score in detection of severe acute pancreatitis.

**Methods:**

This cross sectional study was performed in Emergency department and Surgery department of Dow university hospital from January 2015 to December 2015. A total of 206 patients were included. Those diagnosed with acute pancreatitis on the basis of epigastric pain, serum amylase levels more than 300 (more than 3 times normal) and meeting the inclusion criteria were subjected to investigations for Ranson’s and BISAP scoring. BISAP score was calculated at 24 hours and Ranson’s score both at 24 and 48 hours. A score of > 3 was used to label severe acute pancreatitis according to both scoring systems.

**Results:**

In our study accuracy to predict SAP by BISAP score was 76.2 % and Ranson’s score was 82.2%. On the basis of sensitivity, Ranson’s scores predicted SAP more accurately than BISAP scores (97.4% vs. 69.2%). Regarding specificity, both scores predicted SAP almost equally (78.4% vs. 77.8%).

**Conclusion:**

BISAP score is a valuable tool in predicting severe Acute Pancreatitis in early hours.

## INTRODUCTION

Acute pancreatitis (AP) is a common disorder with substantial burden on the health-care system. AP accounts for 210,000 hospital admissions per annum in the United States.^1^,^2^ Recent studies show the incidence of AP varies between 4.9 and 73.4 cases per 100,000 worldwide.^3^,^4^ An increase in the annual incidence for AP has been observed in most recent studies. It is a complex process in which pancreatic enzyme activation causes local pancreatic damage, resulting in an acute inflammatory response.^5^ The individual patient’s response to pancreatic injury is highly variable and often unpredictable.^5^ Most of the time auto digestion of pancreas is mild and self-limiting but in 20% to 30% cases it develop severe disease that can progress to SIRS, MODS and death.^6^ According to Atlanta classification (2012) severe acute pancreatitis is defined as acute pancreatitis with persistent organ failure more than > 48 hours. A wide range of mortality 20% to 60% has been reported in severe acute pancreatitis.^7^

Many scoring systems have been developed for the detection of severe acute pancreatitis including Ranson’s score, APACHE-II score, CTSI score, MOSS and GLASGOW score.^8^-^10^ The Ranson’s score has been used over three decades. It is moderately accurate in classifying patients in terms of severity, but has the disadvantage of requiring a full 48 hours to be completed, missing a potentially valuable early therapeutic window.^8^,^11^ BISAP score is a newly developed scoring system containing data that can be evaluated at the time of admission which are accurate in predicting patient’s outcome within 24 hours.^12^ The international studies comparing both showed varied results. One of the study shows that the sensitivity of severity acute pancreatitis predicted by BISAP was 74.2%, specificity 68.3% positive predictive value 63.4% and negative predictive value 77.8%^6^ whereas in another study sensitivity is 38.6%, specificity 93.2%, positive predictive value 59.1% and negative predictive value 85.6%.^7^

Ranson’s score requires lots of variables raising cost of complete diagnosis and management whereas BISAP score has less variables which are cost effective and can be done in emergency setting. As the above mentioned studies results are variable, the rationale of this study was to determine BISAP score in emergency setting. If results prove to be in favor of BISAP score than it can help in early diagnosis of severe acute pancreatitis, preventing complications and overall mortality can be reduced.

The objective of this study was to determine the accuracy of BISAP score in comparison with Ranson’s score in detection of severity of acute pancreatitis in a tertiary care hospital.

## METHODS

This cross sectional study was performed in Emergency department and Surgery department of Dow university hospital from January 2015 to December 2015 after the approval from Research and Training monitoring cell of CPSP. A total of 206 patients were included in the study. The sample size was calculated by taking prevalence 25% and 6%. Informed and written consents were taken from all patients. Inclusion criteria contained both males and females, age range between 20 to 50 years, patient presenting within 48 hours of onset of pain (VAS > 5), raised serum amylase levels (more than or equal to 300 IU) within 48 hours of epigastric or upper abdominal pain. Patients who refused to participate in the study, those presenting after 48 hours of onset of pain, patients with other causes of hyperamylasemia and with carcinoma pancreas were excluded from study. Bedside Index of Severity of Acute Pancreatitis (BISAP) was calculated. It includes 5 variables. Blood urea nitrogen levels > 25 mg/dl, impaired mental status or GCS < 15, SIRS, age > 60 years and pleural effusion on imaging. Each variable was granted a score of 1. All patients were subjected to investigations including CBC, LDH, SGOT, RBS, BUN and Chest x-ray. Age SIRS, GCS was recorded to assess the BISAP score and Ranon’s score at 24 hours. Patients were admitted and further evaluated with investigations required for assessment of 48 hours Ranson’s score which included CBC (to asses fall in Hematocrit), BUN, serum calcium, ABGs and fluid deficit. Final agreement between the scores was evaluated at 48 hours. All demographics and outcome variables were entered into the proforma. Outcome variable of this study were age, duration of symptoms, Ranson score and BISAP score. All patients enrolled were diagnosed and classified as mild, moderately severe and SAP according to latest Atlanta classification. SAP was characterized by persistent organ failure for more than 48 hours. A cut off score ≥ 3 was taken to categorize SAP according to Ranson and BISAP score at 24 and 48 hours.

### Statistical Analysis

All data was entered in Statistical Package for Social Science (SPSS) software, Version 16. Mean and standard deviation were calculated for quantitative variables. Frequency and percentages were computed for qualitative variables. Effect modifiers like age, gender, baseline pain were controlled by stratification. Associations between SAP and age and gender were measured using Chi-square analysis.

Further, to see the accuracy for both scoring systems in predicting SAP, Receiver Operating Characteristics (ROC) analysis was also performed. Overall accuracy in predicting SAP, sensitivity, specificity, positive predictive value (+PV), negative predictive value (-PV) were calculated. To check the strength of agreement between standard classified diagnosis using Atlanta classification and Ranson’s and BISAP scores, Kappa coefficient of agreement was also calculated.

To observe the accuracy for both systems, Area under the curves (AUC) with 95% confidence intervals (CI) were also calculated, and curves were plotted against standard diagnosis by Atlanta classification taken as reference line.

All the analyses were performed by using the Statistical Package for Social Science (SPSS) software, Version 16, and Receiver Operating Characteristics (ROC) analysis was performed using software STATA version 8.1. For all analysis p-value < 0.05 was takes as significant.

**Fig. 1:**
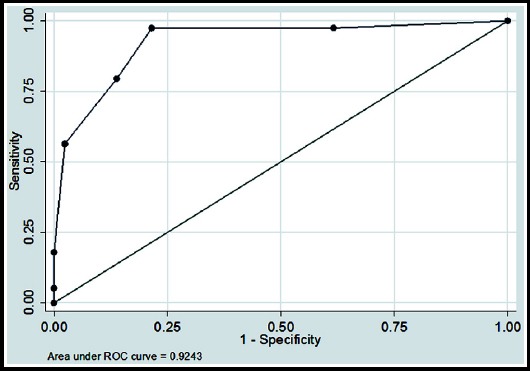
Receiver operation characteristic (ROC) curve of Ranson’s scores in predicting SAP.

## RESULTS

The study included a total of 206 patients of Acute Pancreatitis (AP) from Emergency department and Surgery department of Dow University Hospital on the patient fulfilling the inclusion criteria after taking informed consent. According to Atlanta classification, diagnosed patients with severe AP were found 18.9% (n=39), and patients with mild and moderately severe AP were 81.1% (n=167). Table-I: Describes the characteristics of study participants. The observed BISAP and Ranson’s scores distribution was also reported in Table-I.

**Table I:** Description of patient’s characteristics (n=206).

Study variables	mean ± SD
Age in years	35.25 ± 8.29
Duration of symptoms (hrs.)	10.93 ± 9.94
Baseline pain score (VAS)	6.63 ± 1.31

	*n (%)*

*Severity of pain*	
Moderate	110 (53.4%)
Severe	96 (46.6%)
*Age groups*	
20 - 29	61 (29.6%)
30 - 39	74 (35.9%)
40 - 50	71 (34.5%)
*Sex*	
Male	81 (39.3%)
Female	125 (60.7%)
*BISAP score*	
1	56 (27.2%)
2	86 (41.7%)
3	34 (16.5%)
4	30 (14.6%)
*Ranson’s score*	
1	65 (31.6%)
2	67 (32.5%)
3	20 (9.7%)
4	28 (13.6%)
5-7	26 (12.6%)

BISAP: bedside index of severity in acute pancreatitis

**Fig. 2:**
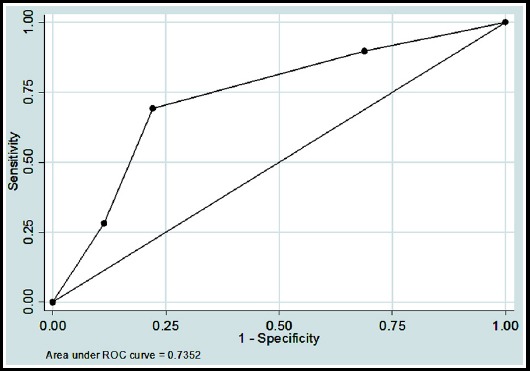
Receiver operation characteristic (ROC) curve of BISAP scores in predicting SAP.

Cutoff of ≥3 score values was taken as criteria for SAP. For BISAP, 31.10% (n=64) patients were found as SAP, whereas 35.90% (n=74) patients had SAP condition according to Ranson’s score. Characteristics of patients with score ≥3 are shown in Table-II. Comparison between Ranson’s and BISAP score in predicting SAP is shown in Table-III.

**Table II:** Characteristics of patients having score ≥ 3 (n=206).

Characteristics	BISAP score ≥ 3		p-value

	No	Yes	

	n (%)	n (%)	
*Age groups*			
20 - 29	35 (24.6)	26 (40.6)	0.017
30 - 39	50 (35.2)	24 (37.5)	
40 - 50	57 (40.1)	14 (21.9)	
*Sex*			
Male	52 (36.6)	29 (45.3)	0.237
Female	90 (63.4)	35 (54.7)	

	Ranson score ≥ 3		*p-value*

	*No*	*Yes*	

	*n (%)*	*n (%)*	

*Age groups*			
20 - 29	24 (18.2)	37 (50.0)	< 0.01
30 - 39	53 (40.2)	21 (28.4)	
40 – 50	55 (41.7)	16 (21.6)	
*Sex*			
Male	43 (32.6)	38 (51.4)	< 0.01
Female	89 (67.4)	36 (48.6)	

Comparison between Ranson’s and BISAP score in predicting SAP is shown in Table-III.

**Table III:** Comparison of BISAP with Ranson’s score in predicting SAP at ≥ 3 (n=206).

	Accuracy	Sensitivity	Specificity	+PV	-PV	AUC (95% CI)	Measure of agreement Kappa (95% CI)
Ranson ≥ 3	82.0%	97.4%	78.4%	51.3%	99.2%	0.92 (0.87 - 0.97)	0.56 (0.44 - 0.67) < 0.001
BISAP ≥ 3	76.2%	69.2%	77.8%	42.2%	91.5%	0.73 (0.64 - 0.82)	0.37 (0.23 - 0.50) < 0.001

BISAP: bedside index of severity in acute pancreatitis, SAP: severe acute pancreatitis, +PV: positive predictive value, -PV: negative predictive value, AUC: Area under curve, CI: confidence intervals

## DISCUSSION

Accurate and quick prediction of SAP is important in order to decrease mortality rate which is around 20% to 60%.^7^,^11^ Many scoring systems have been developed to determine the severe acute pancreatitis early so that better care can be provided to patients and mortality can be decreased. An ideal scoring system should be simple, safe, cheap and less time consuming. BISAP score is one of the newer scoring systems to predict the severity of acute pancreatitis. It has got 5 variable that can be done quickly in emergency department within 24 hours. In this study we determined the accuracy of BISAP score in comparison with Ranson score in predicting the SAP.

Female predominance is noted in most of the studies which is confirmed in our study too^6^,^8^,^13^ whereas no difference is reported between either sex in certain studies.^7^,^14^ Out of these SAP was seen in 54.7% and 48.6% of females according to BISAP and Ranson’s score respectively.

Mean age of patients with acute pancreatitis was 35.25 ± 8.29 years (range 20 – 50) with 35.9% of the population in age range between 30 to 39 years^.^ However majority of the patients fall in 4^th^ and 5^th^ decade of life according to Shabbir S et al^13^ and between 21-30 years according to Khanna AK et al.^6^ No difference of age group was reported by Chen L et al.^7^

In our study, out of 206 patients, total number of 18.9% (n=39) patients has SAP according to Atlanta classification. We found that 38 out of 39 patients had SAP according to Ranson score therefore sensitivity of 97.4% whereas 27 out of 39 patients has SAP according to BISAP score therefore sensitivity of BISAP score was 69.2%. It is comparable to a study conducted by Cho JH et al. according to which sensitivity of Ranson and BISAP score was 85.7 % and 61.9 % respectively.^15^ This is in contrast to BISAP score sensitivity of 58.33% reported by Kim BG et al.^16^

With regard to mortality, 10 patients needed ICU admission, five had sepsis, four with pancreatic necrosis and one with MODS. Out of them there were 6 mortalities and all had Ranson score of > 3 and 4 had BISAP score of > 3.

The accuracy of BISAP score for predicting SAP is 76.2 %. Kappa value is 0.34 showing slight agreement between the the two whereas accuracy of Ranson’s score for predicting SAP is 82.0 % kappa value 0.56 showing fair agreement between the two. This is in comparison with the study reported by Khana AK et al^6^ as in his study accuracy of BISAP and Ranson;s score was 70.8 % and 80.6 % respectively. Slight different results are observed in study conducted by Kim B G et al. in which accuracy of BISAP and Ranson’s score for predicting severe acute pancreatitis was 84% and 94 % respectively which is far greater than accuracy of serum procalcitonin reported in the same study as 58%.^16^

### Limitations of the study

It’s a single center study with small sample size. A multicenter validation study is required to confirm our results and second our observations of BISAP score in severe acute pancreatitis.

## CONCLUSION

BISAP score is a valuable tool in predicting severity of severe acute pancreatitis being simple, easy and cost effective. The assessment is completed in 24 hours that allows early decision making and prompt management. Accuracy of BISAP and Ranson’s score is comparable.
